# Designed Growth of Covalently Bonded WO_3_/PEDOT Hybrid Nanorods Array with Enhanced Electrochromic Performance

**DOI:** 10.3390/ma17133319

**Published:** 2024-07-04

**Authors:** Qing Zhang, Yinhuan Cao, Chuansheng Chen, Xueru Zhang

**Affiliations:** 1 Department of Intelligent Manufacturing, Anhui Vocational and Technical College, Hefei 230011, China; 2 School of Materials Science and Engineering, Hefei University of Technology, Hefei 230009, China; 3 Key Laboratory of Advanced Functional Materials and Devices of Anhui Province, Hefei 230009, China; 4 Department of Architecture and Engineering, Anhui Vocational and Technical College, Hefei 230011, China; 5 Instrumental Analysis Center, Hefei University of Technology, Hefei 230009, China

**Keywords:** tungsten oxide nanorods, poly(3,4-ethylendeioxythiophene), covalently bonded, electrochromism

## Abstract

A covalently bonded WO_3_/PEDOT hybrid nanorods array has been prepared through solvothermal, oil bath, and electrochemical deposition methods using KH57 as a coupling agent. The obtained WO_3_/PEDOT shows substantially increased electrochromic performance with an increased response speed (3.4 s for coloring and 1.2 s for bleaching), excellent optical modulation (86.7% at 633 nm), high coloration efficiency (122.0 cm^2^/C at 633 nm), and distinguished cyclic stability. It was found that the covalent bond interaction between WO_3_ and PEDOT plays an essential role in property enhancement. The covalently bonded inorganic/organic hybrid nanorods array may promise great potential in developing smart-display and energy-efficient materials and devices featuring low energy consumption, cost effectiveness, and environmental protection.

## 1. Introduction

Electrochromic materials have the unique capability to modulate their transmittance, reflectance, and absorbance of solar light in response to a low electric field, displaying reversible variations in color and transparency [1]. Due to their ability to alter coloration and save energy, as well as their environmentally friendly nature, these materials have garnered significant interest for applications in smart windows, static displays, electronic papers, and military camouflage systems [2,3,4,5]. Among inorganic electrochromic materials, nanostructured tungsten oxide (WO_3_) stands out for its ability to provide numerous charge and ion transport channels thanks to its large diffusion coefficient and high specific surface area [6,7,8,9,10]. These attributes endow WO_3_ with high contrast, exceptional optical control, and excellent cycle stability, along with a straightforward preparation process [11,12,13]. However, it suffers from a relatively slow coloring and bleaching speed that restricts its practical applications. In contrast, poly(3,4-ethylendioxythiophene) (PEDOT) is a prototypical cathodically coloring conjugated polymer known for its rapid response time, high coloration efficiency, and robust electrical conductivity [14]. Consequently, the WO_3_/PEDOT composite has the potential to amalgamate the superior properties of both materials, thereby exhibiting enhanced electrochromic performance [13]. Lin et al. prepared WO_3_/PEDOT hybrid nanoparticles using an aqueous suspension method that exhibited an optical contrast of 58.0% and an enhanced coloration efficiency of 84.6 cm^2^/C, compared to 54.2 cm^2^/C for WO_3_ nanoparticle-based electrochromic devices [15]. Yue et al. prepared WO_3_·0.33H_2_O/PEDOT-PB films using a hydrothermal and spin-coating method, exhibiting a much faster response speed than the WO_3_·0.33H_2_O films [16]. Chang-Jian et al. prepared WO_3_/PEDOT nanoparticle films by ink printing, which exhibited superior electrochromic and sun-shielding performance [17]. Eren et al. proposed a one-step approach to assemble a WO_3_/PEDOT electrochromic device that demonstrated good flexibility [18]. Nie et al. prepared WO_3_/PDOT nanorods that showed an improved coloring/bleaching speed and investigated the effect of the conjugated bonds of PEDOT on the properties [19]. Previously, we prepared WO_3_/PEDOT nanorods that showed a high optical contrast of 72% and fast coloring/bleaching switching speed, which were attributed to the synergistic effect between the WO_3_ core and PEDOT shell due to their simultaneously cathodically coloring effect and highly efficient charge transfer [20]. Nevertheless, the effect of the interaction between the WO_3_ and PEDOT on the electrochromic performance has been rarely reported.

Given the auspicious potential of the WO_3_/PEDOT composite for enhancing electrochromic performance, it is imperative to establish a well-considered inorganic/organic interface that fosters positive interactions, thereby optimizing the composite’s electrochromic attributes. The disparate molecular structures of the organic and inorganic components necessitate a strategic approach to interface design [21,22,23,24,25]. Typically, this involves chemically modifying inorganic nanostructures with small organic molecules or leveraging the reactivity of polymer monomers and polymers bearing reactive groups to form covalent bonds at the interface [26]. Such modifications are crucial for enhancing electrical conductivity within the nanocomposites and facilitating the ion transport process, which, in turn, upgrades the electrochemical and electrochromic performance of the nanocomposite material [27,28,29]. Inorganic nanostructures can undergo chemical modifications to form conductive polymers through the formation of covalent bonds with substances [30,31]. For instance, Xiong and colleagues have synthesized a range of nanocomposites featuring covalently bonded polyaniline paired with carbon nanotubes, fullerenes, and acetyl ferrocene [32,33,34], all exhibiting enhanced electrochromic properties. Similarly, Yang and coworkers have developed covalently bonded WO_3_/polyvinylimidazole core–shell microspheres that showcase improved chemical stability and electrochromic performance [35]. Despite these advancements, reports on the synthesis of covalently bonded WO_3_/PEDOT composites are scarce.

In this study, we chemically modified WO_3_ using γ-methacryloxypropyl trimethoxy silane (a silane coupling agent, KH570) to create a platform for covalent bonding. Subsequently, we fabricated covalently bonded WO_3_/PEDOT nanowire array films through a combination of solvothermal, oil bath, and electrodeposition techniques. This covalent bonding enhances electron conduction and ion transport within the nanocomposites, thereby significantly improving their electrochromic properties.

## 2. Materials and Methods

### 2.1. Fabrication and Chemical Modification of WO_3_ Nanorod Array Film

WO_3_ nanorod array film was fabricated by combining magnetron sputtering and solvothermal methods. A WO_3_ seed layer was deposited on an FTO glass substrate using WO_3_ (purity: 99.99%) as a target at room temperature by radio frequency magnetron sputtering method. During sputtering, high-purity argon and oxygen flow were introduced in a ratio of 1:1. The sputtering power was kept at 50 W at a pressure of 0.8 Pa and a deposition time of 10 min. After the sputtering process, the FTO conductive glass substrate was removed and annealed at 400 °C for two hours in a three-zone tubular furnace to obtain the WO_3_ seed layer required for the solvothermal reaction.

The solvothermal method was used to prepare WO_3_ nanorods array. First, 2.5 g of tungstic acid powder was dissolved in a mixture of 50 mL deionized water and 30 mL of a 30% weight solution of hydrogen peroxide to obtain a clear solution after heating and stirring at 95 °C. The solution was then diluted with deionized water to 200 mL, resulting in a molar concentration of 0.05 M tungstic acid precursor solution. Next, 21 mL of the prepared 0.05 M tungstic acid precursor solution was taken and mixed with 3.5 mL of 3 M hydrochloric acid solution, 26 mL of deionized water, and 13 mL of acetonitrile. After thorough mixing, the mixture was placed into a high-pressure autoclave with a polytetrafluoroethylene (PTFE) liner. The autoclave was sealed and heated to 180 °C at a rate of 8 °C/min and maintained at this temperature for 12 h. After the reaction, the autoclave was naturally cooled to room temperature and the FTO conductive glass substrate was taken out and thoroughly washed with deionized water and dried at 60 °C for 1 h to obtain WO_3_ nanorods array on the FTO substrate.

Chemical modification of the WO_3_ (C-WO_3_) nanorod array film adopted oil bath method with γ-methacryloxypropyl trimethoxy silane (silane coupling agent, KH570). The pH value of the mixed solution of deionized water (9 mL) and anhydrous alcohol (27 mL) was adjusted to 4 with 3 M HCl, and KH570 (9 mL) was added dropwise into the above solution while stirring. The F-doped SnO_2_ (FTO) conducting glass that grew WO_3_ nanorod array film was fixed vertically with tape in 50 mL beaker that was equipped with the final solution, and then they were put into an oil bath pot that was kept for 5 h at 70 °C under 30 rpm stirring speed. Subsequently, the FTO conducting glass was taken out, maintained for 12 h in anhydrous alcohol, and then washed several times with deionized water and dried at room temperature, and the C-WO_3_ nanorod array film was finally obtained.

### 2.2. Fabrication of C-WO_3_/PEDOT Core/Shell Nanorod Array Film

The fabrication of C-WO_3_/PEDOT was conducted in a three-electrode system, in which the platinum wire, the Ag/AgCl electrode, and the C-WO_3_ nanorod array film on FTO conducting glass were used as the counter-electrode, the reference electrode, and the working electrode, respectively. The electrolyte was made by dispersing 0.1 M 3,4-ethoxylene dioxy thiophene (EDOT) and 0.2 M LiClO_4_ in propylene carbonate (PC) via ultrasonication for 30 min. Electrochemical polymerization was carried on for 8 s at 2.0 mA/cm^2^ without stirring, and the potential was kept between +0.86 V and +0.91 V vs. Ag/AgCl reference electrode during the electrodeposition process. Finally, C-WO_3_/PEDOT hybrid nanorod array film was received. WO_3_/PEDOT hybrid nanorod array film was fabricated for comparison using the same process and parameters as those of C-WO_3_/PEDOT except that the WO_3_ film was not chemically modified with KH570.

### 2.3. Characterization

The morphology and structure of film samples were characterized by field emission scanning electron microscope (FESEM, SU8020, Hitachi Ltd., Tokyo, Japan), high-resolution transmission electron microscopy (HRTEM, JEM-2100F, JEOL Ltd., Tokyo, Japan), X-ray diffractometer (CuKα radiation, λ = 0.15418 nm, D/MAX2500V, Rigaku Corporation, Tokyo, Japan), X-ray photoelectron spectroscope (XPS, ESCALAB 250Xi, Thermo Scientific, Shanghai, China), confocal micro-Raman spectrometry (LabRAM HR Evolution, HORIBA JOBIN YVON, Palaiseau, France), and Fourier transform infrared spectrometer (FTIR, Nicolet IS50/In10, Thermo Scientific, Shanghai, China). Electrochemical and electrochromic properties of film samples were tested by electrochemical workstation (CHI760E, Chenhua, China) and UV-VIS-NIR spectrophotometer (UV-3600, Shimadzu Corporation, Tokyo, Japan) in a three-electrode system with 1 M LiClO_4_/PC (propyl carbonate) solution as electrolyte. In the three-electrode system, the platinum wire, the Ag/AgCl electrode, and the film on FTO conducting glass were used as the counter-electrode, the reference electrode, and the working electrode, respectively. The transmittance spectra were obtained with UV-3600 by applying −1.0 V and 1.0 V voltages in the spectral range of 400–800 nm and the dynamic optical transmittance was measured at 633 nm through UV-3600 together with CHI760E by applying −1.0 V and 1.0 V voltages for 20 s, respectively.

## 3. Results

### 3.1. Morphology and Structure

**Figure 1:**
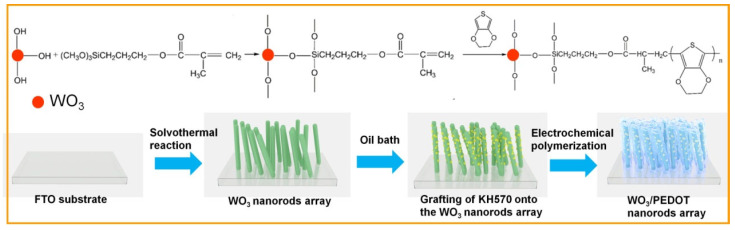
Illustration for the chemical modification and preparation of the C-WO_3_/PEDOT nanorod array film.

Figure 1 shows the schematic diagram of synthesizing WO_3_/PEDOT nanorods arrays and the mechanism of the chemical modification of KH570 onto the nanorods. The WO_3_ nanorods array was chemically modified by KH570 in an acid environment through a high-temperature condensation reaction. The methoxy groups of KH570 hydrolyze with the hydroxyl groups of the WO_3_ nanorod array, in which KH570 formed a covalent bond with the WO_3_ nanorod array (C-WO_3_). The methacrylate groups on the WO_3_ nanorod array can further react with the 3,4-ethoxylrnr dioxy thiophene (EDOT) monomer through radical polymerization; finally, a covalently bonded WO_3_/PEDOT nanorod array is gained.

**Figure 2:**
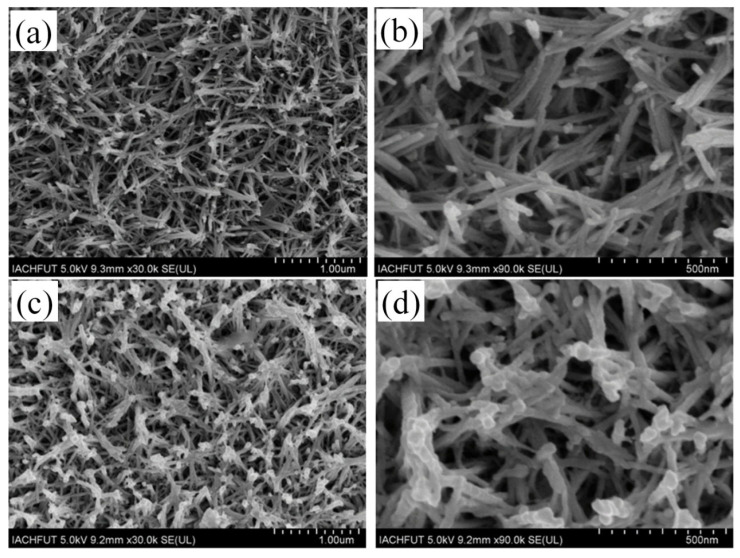
FESEM images of (**a**,**b**) WO_3_/PEDOT nanorod array film; (**c**,**d**) C-WO_3_/PEDOT nanorod array film.

Figure 2 illustrates the morphological differences in the WO_3_/PEDOT nanorod arrays before and after interface functionalization as characterized by field emission scanning electron microscopy (FESEM). Figure 2a,b display the FESEM images of the WO_3_/PEDOT arrays prior to KH570 modification. The low-magnification image in Figure 2a reveals the free-standing and uniform growth of the nanorods. The high-magnification image in Figure 2b shows that the diameter of the nanorods ranges from 15 to 50 nm. Following the chemical modification with KH570 to form covalent bonds, the C-WO_3_/PEDOT nanorod arrays were obtained, as seen in Figure 2c,d. The surface morphology of the C-WO_3_/PEDOT nanorod arrays appears rougher and more vertically aligned on the surface of the FTO conductive glass, with diameters approximately ranging from 20 to 60 nm, which are thicker than the unmodified WO_3_/PEDOT nanorods. This suggests that the high reactivity of KH570 may facilitate the growth of PEDOT. Such a morphological characteristic may provide a large specific surface area, which is beneficial for the transmission of ionic electrons and electrochemical reaction dynamics [36,37].

**Figure 3:**
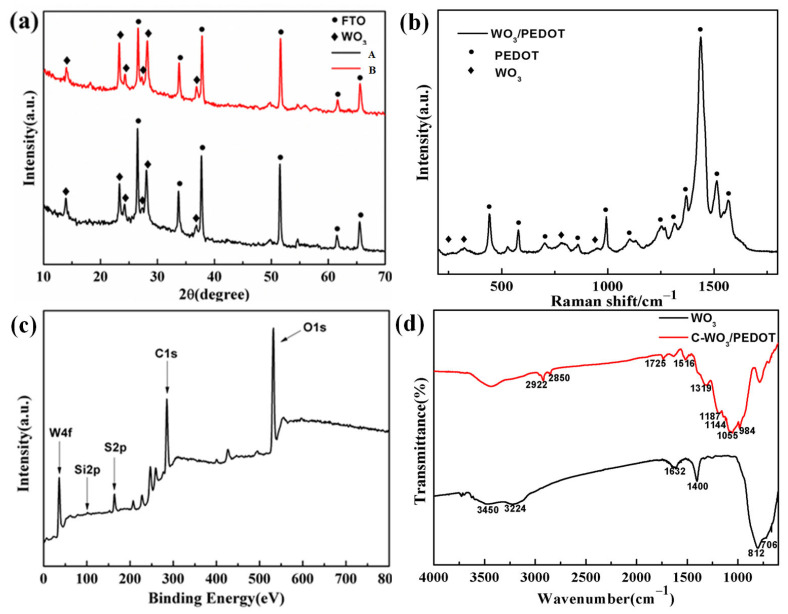
(**a**) XRD pattern of (**a**) WO_3_/PEDOT and C-WO_3_/PEDOT hybrid nanorods array film. (**b**) Raman spectrum, (**c**) XPS survey, and (**d**) FTIR spectrum of C-WO_3_/PEDOT hybrid nanorods array.

To ascertain the phase structure of the nanorods and assess the impact of chemical modification, X-ray diffraction (XRD) was performed on the WO_3_/PEDOT nanorod arrays with and without KH570 modification, as depicted in Figure 3a. Besides the peaks corresponding to the FTO glass, the other diffraction peaks are assignable to the hexagonal WO_3_ phase (PDF No. 85–2460). Comparing the XRD pattern of the WO_3_/PEDOT (plot A) with that of the C-WO_3_/PEDOT (plot B), it is evident that both the degree of crystallinity and the interplanar spacing remain largely unchanged, suggesting that the WO_3_ phase structure is not altered by the KH570 chemical modification.

To confirm the presence of the PEDOT layer on the WO_3_ nanorods, Raman spectroscopy was employed to obtain fingerprint information of the samples, as shown in Figure 3b, which can display the characteristic information of WO_3_ and PEDOT based on the characteristic bands. In the Raman spectrum, the characteristic peaks located at 258 cm^−1^ and 301 cm^−1^ correspond to hexagonal WO_3_, and the broad peak at 776 cm^−1^ is attributed to the fitting of three characteristic peaks at 697 cm^−1^, 787 cm^−1^, and 820 cm^−1^, respectively. The peaks at 697 cm^−1^ and 820 cm^−1^ belong to the metastable hexagonal phase of WO_3_, the peak at 787 cm^−1^ is attributed to hydrated WO_3_, indicating that the WO_3_ nanorods array contains hydrated WO_3_, and the peak at 952 cm^−1^ can be attributed to the stretching mode of the terminal W=O bond [38]. In the Raman spectrum, the characteristic peaks located at 442 cm^−1^, 577 cm^−1^, and 993 cm^−1^ are attributed to the ring deformation vibration of thiophene; the characteristic peak at 1131 cm^−1^ can be attributed to the C-O-C deformation vibration; the symmetric stretching mode corresponding to C_α_-C_α_ is attributed to the peak at 1254 cm^−1^; the peaks located at 1367 cm^−1^, 1436 cm^−1^, and 1512 cm^−1^ correspond to the stretching deformation of C_β_-C_β_, the symmetric stretching vibration of C_α_=C_β_, and the asymmetric vibration of C_α_=C_β_, respectively [39,40,41]. The Raman spectrum of the WO_3_/PEDOT nanowire array includes the characteristic bands of WO_3_ and PEDOT, and the comprehensive TEM results can prove that the PEDOT shell is uniformly wrapped around the WO_3_ core layer. Given the sensitivity of Raman spectroscopy to sample surfaces, the stronger vibration peaks corresponding to the PEDOT layer are more pronounced than those of the WO_3_ core.

The composition and chemical state of the sample were further characterized using X-ray photoelectron spectroscopy (XPS), as presented in Figure 3c. The major peaks at 532.3 eV and 284.9 eV are assigned to O1s and C1s, respectively. Additionally, the peak at 101.5 eV can be attributed to Si2p from KH570, confirming the presence of the coupling agent.

To examine the surface functionalization of the samples in detail, Fourier transform infrared (FTIR) spectroscopy was utilized, and the resulting spectra of WO_3_ and C-WO_3_/PEDOT nanorod array films are displayed in Figure 3d. In the FTIR spectrum of WO_3_, the peaks at approximately 3450, 3224, and 1632 cm^−1^ are associated with OH stretching vibration and the stretching and bending vibrations of absorbed water, while the peak at 1400 cm^−1^ is an unusual value for OH stretching and bending vibrations. The peaks at 706 and 813 cm^−1^ are attributed to the shortening of W–O bonds in hexagonal WO_3_ [42]. In the FTIR spectrum of C-WO_3_/PEDOT, the peaks at around 2922 and 2850 cm^−1^ are indicative of methylene group stretching vibrations, and the peak at 1725 cm^−1^ arises from the C=O stretching vibration of KH570 [43,44]. The peaks at approximately 1055, 1144, and 1187 cm^−1^ are due to C-O-C bond stretching in the ethylene dioxy group. The peaks at 1319 and 1516cm^−1^ are assigned to the C-C or C=C stretching of the quinoid structure of the thiophene ring and stretching of the thiophene ring, respectively. The vibration at 984 cm^−1^ corresponds to the C-S bond in the thiophene ring, confirming the presence of PEDOT [45].

Collectively, the Raman, XPS, and FTIR results suggest that PEDOT has been successfully chemically attached to the surface of the WO_3_ nanorod arrays. Based on these findings, it can be deduced that the covalently bonded WO_3_/PEDOT nanorod arrays were obtained through the use of KH570. To present the sample’s fingerprint information more distinctly, the characteristic peaks and their corresponding assignments from the Raman and FTIR data are summarized in Table S1 of the Supplementary Material.

**Figure 4:**
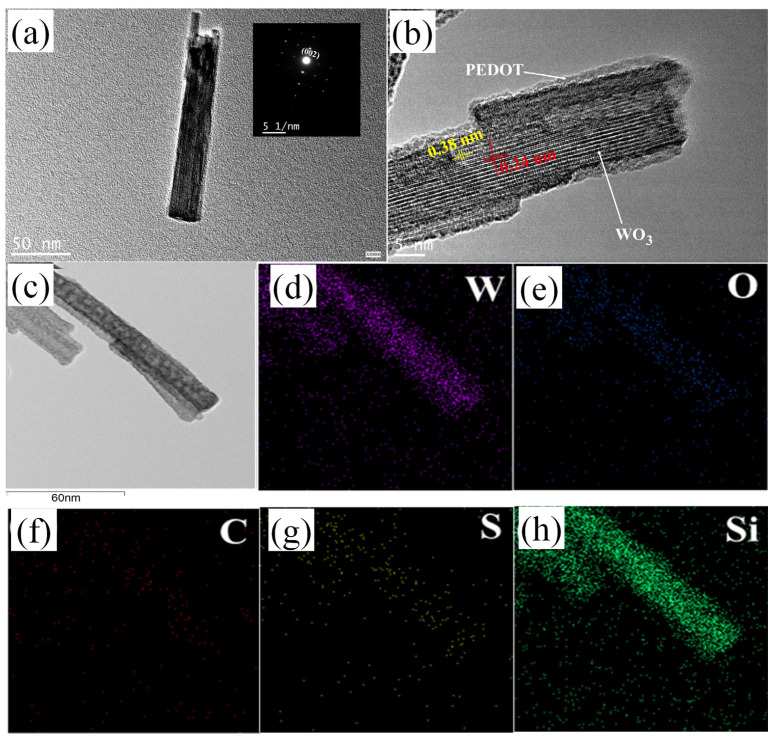
(**a**) Bright-field TEM image (inset: SAED), (**b**) HRTEM characterization, and EDX mapping results, including (**c**) morphology image, (**d**) tungsten, (**e**) oxygen, (**f**) carbon, (**g**) sulfur, and (**h**) silicon element distribution of the C-WO_3_/PEDOT nanorod arrays.

To obtain insight into the microstructure of the hybrid nanorods, high-resolution transmission electron microscopy (HRTEM) and energy-dispersive X-ray spectroscopy (EDX) mapping were conducted as shown in Figure 4. Figure 4a presents a typical bright-field image of a C-WO_3_/PEDOT nanorod, indicating that the diameter of the nanorod array is about 25 nm. The inserted selected area electron diffraction (SAED) pattern exhibits bright diffraction spots, indicating the single-crystal nature of the nanorod. Figure 4b shows an HRTEM image of the C-WO_3_/PEDOT nanorod, displaying a crystalline core/amorphous shell structure. The adjacent lattice spacings of 0.38 nm and 0.24 nm in the crystalline core correspond to the (002) and (210) planes of the hexagonal WO_3_ phase, respectively. The (002) plane is perpendicular to the longitudinal axis of the nanorod, suggesting that the nanorod grows preferentially along the [002] direction, which is consistent with the SAED determination. The amorphous shell is presumed to be the PEDOT layer, and energy-dispersive X-ray (EDX) mapping was conducted to confirm this. As shown in Figure 4c–h, the EDX mapping results indicate that the core/shell structure is composed of W, O, Si, C, and S elements. W and O are detected in the core from the WO_3_ nanorods, while the Si element is derived from KH570 and is distributed uniformly across the nanorods. The C and S elements are primarily localized at the position of the shell structure, which can be attributed to the thin PEDOT layer.

### 3.2. Electrochemical and Electrochromic Properties

**Figure 5:**
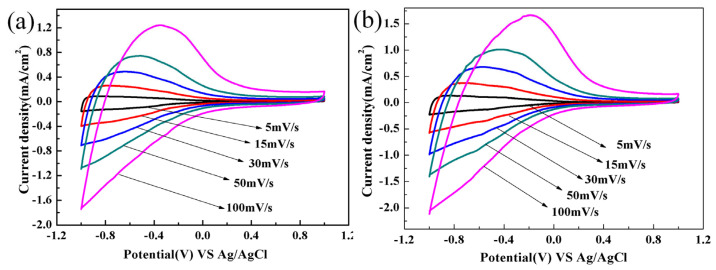
Cyclic voltammetry curves of (**a**) WO_3_/PEDOT and (**b**) C-WO_3_/PEDOT nanorod array film tested in 1.0 M LiClO_4_/PC solution with scanning rate of 5, 15, 30, 50, 100 mV/s.

To delve into the electrochemical reaction processes of the WO_3_/PEDOT and C-WO_3_/PEDOT nanorod arrays, cyclic voltammetry (CV) measurements were conducted at various scan rates ranging from 5 to 100 mV/s in a 1.0 M LiClO_4_/PC electrolyte solution. As depicted in Figure 5, pronounced anodic and cathodic peaks are evident, corresponding to the extraction and insertion of lithium ions and electrons from WO_3_, respectively.

As the scan rate increases, the oxidation peaks are seen to shift toward more positive potentials while the reduction peaks shift toward more negative potentials, suggesting that the electrochemical process is diffusion-controlled. When comparing the WO_3_/PEDOT nanorod arrays (Figure 5a) with the C-WO_3_/PEDOT nanorod arrays (Figure 5b), it is apparent that the C-WO_3_/PEDOT arrays exhibit a higher exchange current density and a larger area within the voltammograms. This indicates that the C-WO_3_/PEDOT arrays possess a greater number of active sites and facilitate more effective oxidation and reduction processes.

These enhancements can be attributed to the larger specific surface area, the increased number of shorter ion and electron transport channels, and the reduced internal resistance of the C-WO_3_/PEDOT nanorod arrays. These factors contribute to the improved electrochemical performance observed in the C-WO_3_/PEDOT system.

**Figure 6:**
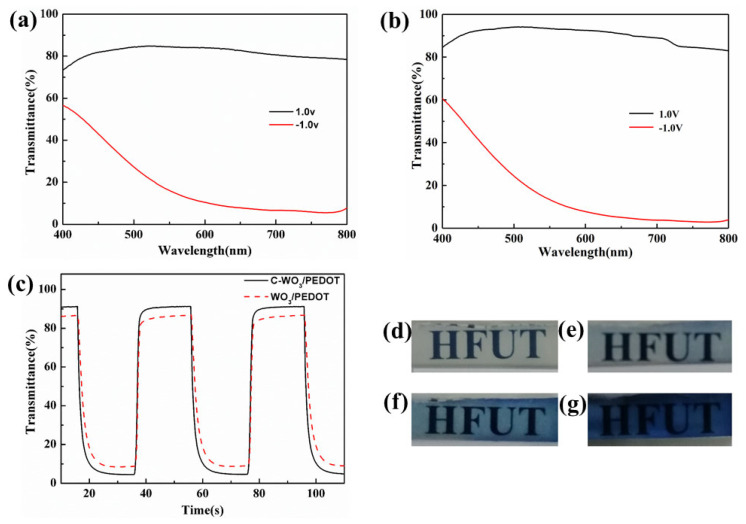
Transmittance spectra of (**a**) WO_3_/PEDOT, (**b**) C-WO_3_/PEDOT, and (**c**) dynamic optical transmittance of WO_3/_PEDOT, and C-WO_3_/PEDOT nanorod array film. (**d**–**g**) Digital photographs of nanorod array film measured at the voltages of 0 V, −0.5 V, −1.0 V, and −1.5 V.

In Figure 6a,b, the transmittance spectra of the WO_3_/PEDOT and C-WO_3_/PEDOT nanorod array films in both the colored (T_c_ blue) and bleached (T_b_ transparent) states are depicted. These spectra were obtained by applying −1.0 V and 1.0 V voltages, respectively, within the spectral range of 400–800 nm using a 1.0 M LiClO_4_/PC solution as the electrolyte. Upon analysis, the maximum optical modulation (ΔT = T_b_ − T_c_) was observed at a wavelength of 633 nm, with the C-WO_3_/PEDOT nanorod array film exhibiting a broader modulation range than its WO_3_/PEDOT counterpart. The dynamic optical transmittance at 633 nm, as shown in Figure 6c, was measured by applying −1.0 V and 1.0 V voltages for 20 s in a 1.0 M LiClO_4_/PC solution. The kinetic process illustrates that the C-WO_3_/PEDOT nanorod array film demonstrates a faster response time compared to the WO_3_/PEDOT film, attributed to the enhanced ability of the C-WO_3_/PEDOT film to facilitate the rapid insertion/extraction of ions and electrons. Figure 6d–g are digital photographs of the C-WO_3_/PEDOT nanorod array films at applied voltages of 0 V, −0.5 V, −1.0 V, and −1.5 V, respectively. These images underscore the capability of the composite nanostructure to modulate its color depth in response to the applied voltage.

Table 1 provides a comprehensive summary of the electrochromic performance data corresponding to the observations presented in Figure 6a–g.

Coloration efficiency (CE) is a critical parameter used to assess the performance of electrochromic materials. It quantifies the change in optical density (ΔOD) per unit charge density (Q/A) introduced into the material and can be calculated using the following equation: CE = ΔOD/(Q/A). ΔOD is calculated as log (T_b_/T_c_), where T_b_ and T_c_ represent the transmittance in the bleached and colored states, respectively.

**Figure 7:**
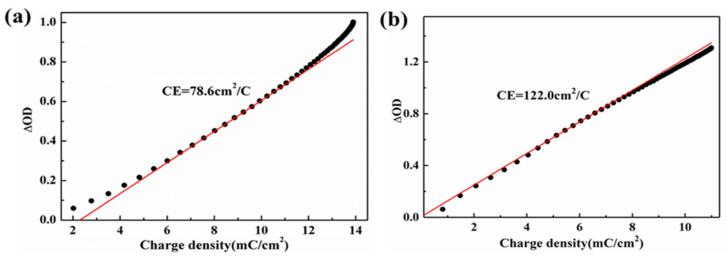
The relationship between optical density and charge density for the WO_3_/PEDOT (**a**) and C-WO_3_/PEDOT (**b**) nanorods array at a wavelength of 633 nm and an applied voltage of −1.0 V.

Figure 7 illustrates the relationship between optical density and charge density for the WO_3_/PEDOT and C-WO_3_/PEDOT nanorod array films at a wavelength of 633 nm and an applied voltage of −1.0 V. The slope of the linear region of the curve was determined, yielding CE values of 78.6 cm^2^/C for WO_3_/PEDOT and 122.0 cm^2^/C for C-WO_3_/PEDOT. The higher CE value indicates that the material can provide a greater optical contrast with minimal changes in the amount of ion or electron insertion/extraction, thus significantly enhancing the stability of the cycle.

The increased CE value of the covalently bonded WO_3_/PEDOT nanorod array is primarily due to the rough surface of the nanorod array, which provides a larger specific surface area, a higher density of active sites, more rapid ion and electron transport channels, and reduced resistance for electrochemical reactions. These improvements contribute to the superior electrochromic performance observed in the C-WO_3_/PEDOT system.

Cyclic stability is a critical factor in the practical application of electrochromic materials. To assess the cyclic stability of the WO_3_/PEDOT and C-WO_3_/PEDOT nanorod array films in a 1.0 M LiClO_4_/PC solution, cyclic voltammetry (CV) with a square-wave potential of −1.0 V (20 s) and +1.0 V (20 s) was employed. Each cycle consisted of one coloring time (20 s) and one bleaching time (20 s), totaling 40 s.

**Figure 8:**
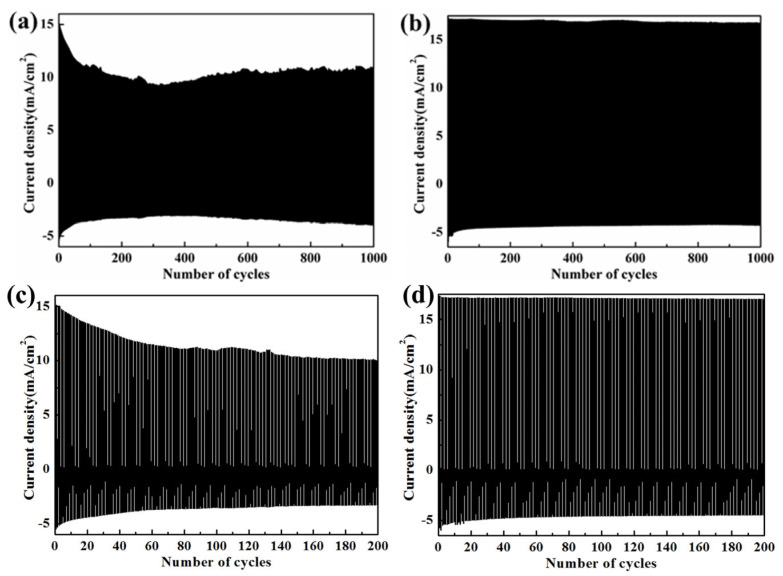
Chronoamperometry measurements of (**a**) WO_3_/PEDOT, (**b**) C-WO_3_/PEDOT nanorod array film for 1000 cycles with a potential between −1.0 V (20 s) and 1.0 V (20 s), and (**c**) a close-up view of the curve for the initial 200 cycles in Figure 8a, (d) a close-up view of the curve for the initial 200 cycles in Figure 8b.

As depicted in Figure 8, the electrochemical current pulse of the C-WO_3_/PEDOT nanorod array film (Figure 8b) exhibits stable performance, maintaining 90.5% of the initial optical contrast after 1000 cycles, while that of the WO_3_/PEDOT nanorods (Figure 8a) decreases to 75.6%. Figure 8c,d show an enlarged perspective of the initial 200 cycles depicted in Figure 8a,b, respectively. Figure 8c reveals a pronounced decline in current density during cycling for the WO_3_/PEDOT system. This decrement signifies structural degradation. By contrast, Figure 8d demonstrates superior stability of the current density. The enhanced cyclic stability of the c-WO_3_/PEDOT nanorod array can be attributed to the covalent bond interaction between WO_3_ and PEDOT, which arises from the chemical modification of the WO_3_ nanorod array with KH570. This covalent bonding enhances the structural integrity and stability of the nanorod array, contributing to its improved electrochromic performance.

Table 2 encapsulates recent studies on WO_3_/PEDOT electrochromic films, delineating key performance metrics such as optical contrast, coloring/bleaching time, and coloration efficiency. The data indicate that the covalently bonded WO_3_/PEDOT nanorod array film obtained in our work exhibits superior electrochromic performance relative to those reported in the recent literature.

## 4. Conclusions

The covalently bonded WO_3_/PEDOT nanorod array film has been successfully prepared by oil bath, solvothermal, and electrodeposition methods, in which PEDOT is covalently bonded to a WO_3_ nanorod array with the chemical modification of KH570. The covalently bonded WO_3_/PEDOT nanorod array has a larger specific surface area, more active units, more rapid transport channels, and a covalent bond interaction between WO_3_ and PEDOT; therefore, the results display enhanced electrochemical and electrochromic properties and cyclic stability. This kind of covalently bonded inorganic/organic hybrid nanostructure composite with enhanced electrochromic properties has broad development space in the development of electrochromic materials and the application of electrochromic devices.

## Figures and Tables

**Table 1:** Electrochromic performance data of WO_3_ nanorod array film, PEDOT film, WO_3_/PEDOT hybrid nanorods array film, and C-WO_3_/PEDOT hybrid nanorods array film.

Samples	Optical Modulation Range (%) 633 nm	Coloring Time (s) t_c_	Bleaching Time (s) t_b_
WO_3_	73.0	9.4	6.0
PEDOT	34.4	0.3	0.1
WO_3_/PEDOT	78.2	4.6	2.0
c-WO_3_/PEDOT	86.7	3.4	1.2

**Table 2:** Overview of several recent studies on WO_3_/PEDOT electrochromic films.

	Optical Contrast (%)	Coloring Time (s)	Bleaching Time (s)	Coloration Efficiency (cm^2^C^−1^)	Reference
WO_3_/PEDOT nanoparticles	58.0	/	/	84	[15]
WO_3_·0.33H_2_O/PEDOT films	50.9	32	12	74.6	[16]
WO_3_/PEDOT printing films	54.1	1.2	1.1	83.87	[17]
WO_3_/PEDOT powders	38.7	6.44	5.33	/	[18]
WO_3_/PEDOT inverse opal films	52.0	6.7	5.8 s	/	[25]
WO_3_/PEDOT nanowires	68.2	22.4	26.0	109.9	[19]
WO_3_/PEDOT nanowires	72.0	3.8	3.6	163.5	[20]
WO_3_/PEDOT nanowires	86.7	3.4	1.2	122.0	This work

## Data Availability

All the data are contained within the article.

## Supplementary Materials

The following are available online at https://www.mdpi.com/article/10.3390/ma17133319/s1, Table S1: Characteristic peaks and corresponding assignments from Raman and FTIR data of the C-WO_3_/PEDOT nanorods.
