# Supplementary material for: Designed Growth of Covalently Bonded WO3/PEDOT Hybrid Nanorods Array with Enhanced Electrochromic Performance
Source: Materials (Basel). 2024 Jul 4;17(13):3319. doi: 10.3390/ma17133319 (PMC11243032; doi:10.3390/ma17133319)
Supplement: Supplementary file 1 [file materials-17-03319-s001.zip › materials-3064269-supplementary.pdf]

Table S1. Characteristic peaks and corresponding assignments from Raman and FTIR data of the C-WO_3/PEDOT nanorods

Raman shift(cm^{-1})	assignment	FITR spectrum(cm^{-1})	assignment
258 , 301	hexagonal WO3	3450	OH stretching vibration
697, 820	metastable hexagonal WO3	3224	stretching vibration of absorbed water
787	hydrated WO3	1632	bending vibrations of absorbed water
952	stretching mode of W=O bond	706 , 813	shortening of W–O bonds
442, 577 , 993	ring deformation vibration of thiophene	2922, 2850	stretching vibrations of methylene
1131	C-O-C deformation vibration	1725	C=O stretching of KH570
1254	symmetric stretching mode of Cα-Cα	1055, 1144 1187	C-O-C stretching of ethylene dioxy
1367	stretching deformation of Cβ-Cβ,	1319	C-C stretching of the thiophene ring
1436	symmetric stretching vibration of Cα=Cβ,	1516	C=C stretching of the thiophene ring
1512	asymmetric vibration of Cα=Cβ,	984	C-S bond of the thiophene ring
